# BORIS in Hormone-Driven Cancers: Epigenetic Reprogramming, Transcriptional Plasticity, and Therapeutic Implications

**DOI:** 10.3390/cells15212001

**Published:** 2026-11-03

**Authors:** Sara Saleem, Luke Gaughan, Craig N. Robson

**Affiliations:** 1 Newcastle University Centre for Cancer, Paul O’Gorman Building, Newcastle upon Tyne NE2 4AD, UK; luke.gaughan@newcastle.ac.uk; 2 Faculty of Biomedical Sciences, University of Benghazi, Benghazi, Libya

**Keywords:** BORIS, epigenetic reprogramming, endocrine therapy resistance, cancer-testis antigen, chromatin remodelling, hormone-driven cancers, androgen receptor

## Abstract

**Highlights:**

**What are the main findings?**

- BORIS, a germline-restricted paralogue of CTCF, is activated in ovarian, endometrial, breast and prostate cancers.
- BORIS binds CTCF sites, displacing CTCF or co-occupying them with it, and can reprogram clustered sites into alternative transcriptional start sites through SRCAP-mediated H2A.Z deposition.

**What are the implications of the main findings?**

- BORIS may contribute to hormone receptor loss and endocrine therapy resistance, but direct functional evidence in breast, endometrial and prostate cancers is still lacking.
- As a cancer-testis antigen with a distinct chromatin mechanism, BORIS is a candidate biomarker and immunotherapy target in hormone-driven cancers.

**Abstract:**

Hormone-driven cancers, including prostate, breast, endometrial and ovarian cancers, are a leading cause of cancer-related mortality. Endocrine therapies targeting androgen receptor and oestrogen receptor/progesterone receptor signalling have improved outcomes, but acquired resistance arising through genetic and epigenetic mechanisms that bypass receptor dependence remains a major challenge. BORIS (Brother of the Regulator of Imprinted Sites), a CTCF paralogue, is a germline-restricted regulator that becomes aberrantly reactivated in cancer through promoter hypomethylation, p53 loss, and loss of CTCF-mediated repression. Once expressed, BORIS occupies CTCF sites, displacing or co-occupying them with CTCF, and reprograms clustered sites into alternative transcriptional start sites through SRCAP-mediated H2A.Z deposition, activating germline genes that sustain proliferation, and therapeutic resistance. BORIS expression correlates with poor prognosis in ovarian and endometrial cancers, associates with ER/PR levels in breast cancer, and correlates with Gleason score in prostate cancer. BORIS represses AR signalling in ovarian cancer and inversely correlates with receptor expression in endometrial cancer. Its cancer-testis antigen properties position BORIS as an immunotherapy target, with preclinical vaccine efficacy demonstrated in breast cancer models. This review synthesises the disease-specific evidence positioning BORIS as a driver of transcriptional reprogramming and endocrine resistance, identifies current knowledge gaps, and discusses translational implications of targeting this pathway.

## 1. Introduction

Hormone-dependent cancers, principally prostate, breast, endometrial and ovarian cancers, represent one of the most significant sources of oncological morbidity and mortality worldwide. According to GLOBOCAN 2022 estimates, breast and prostate cancers rank as the second and fourth most frequently diagnosed malignancies globally, accounting for 11.6% and 7.3% of all new cancer cases, respectively [1]. Androgenic and oestrogenic hormones are key drivers of the initiation, development, and progression of these hormone-responsive tumours. Specifically, the androgen receptor (AR) plays a central role in prostate cancer, while the oestrogen receptor (ER) and progesterone receptor (PR) contribute to breast and gynaecological malignancies [2].

Steroid hormone signalling has been a therapeutic target in hormone-responsive cancers for decades. Endocrine interventions, including androgen deprivation therapy (ADT), AR antagonists, anti-oestrogen therapy, aromatase inhibitors, and selective ER degraders (SERDs), have significantly improved outcomes in patients in these hormone-sensitive diseases [3,4]. However, acquired resistance to endocrine therapy remains the major clinical challenge across this group of malignancies. In prostate cancer, progression from androgen-sensitive disease to castration-resistant prostate cancer (CRPC) occurs in the majority of patients [5], and a subset of these tumours undergo lineage switching to a neuroendocrine phenotype that is refractory to AR-directed treatment [6]. Similarly, for ER-positive breast cancer, approximately 20–40% of patients with stage I-III disease will experience relapse despite endocrine therapy, reflecting the emergence of either primary or acquired resistance [7].

Resistance to endocrine therapy arises through multiple genetic mechanisms, including somatic mutations in steroid receptor ligand-binding domains, gene amplification, alternative splicing, and the activation of bypass signalling pathways. While these mechanisms are well-established and clinically relevant, they do not fully account for the observed resistance landscape. Non-mutational epigenetic-reprogramming has now emerged as a hallmark of cancer, with DNA methylation, histone modifications and chromatin remodelling all driving cancer progression and therapeutic resistance [8]. BORIS (Brother of the Regulator of Imprinted Sites), also known as CTCFL (CTCF-like), is a key contributor to this epigenetic resistance landscape.

**Figure 1:**
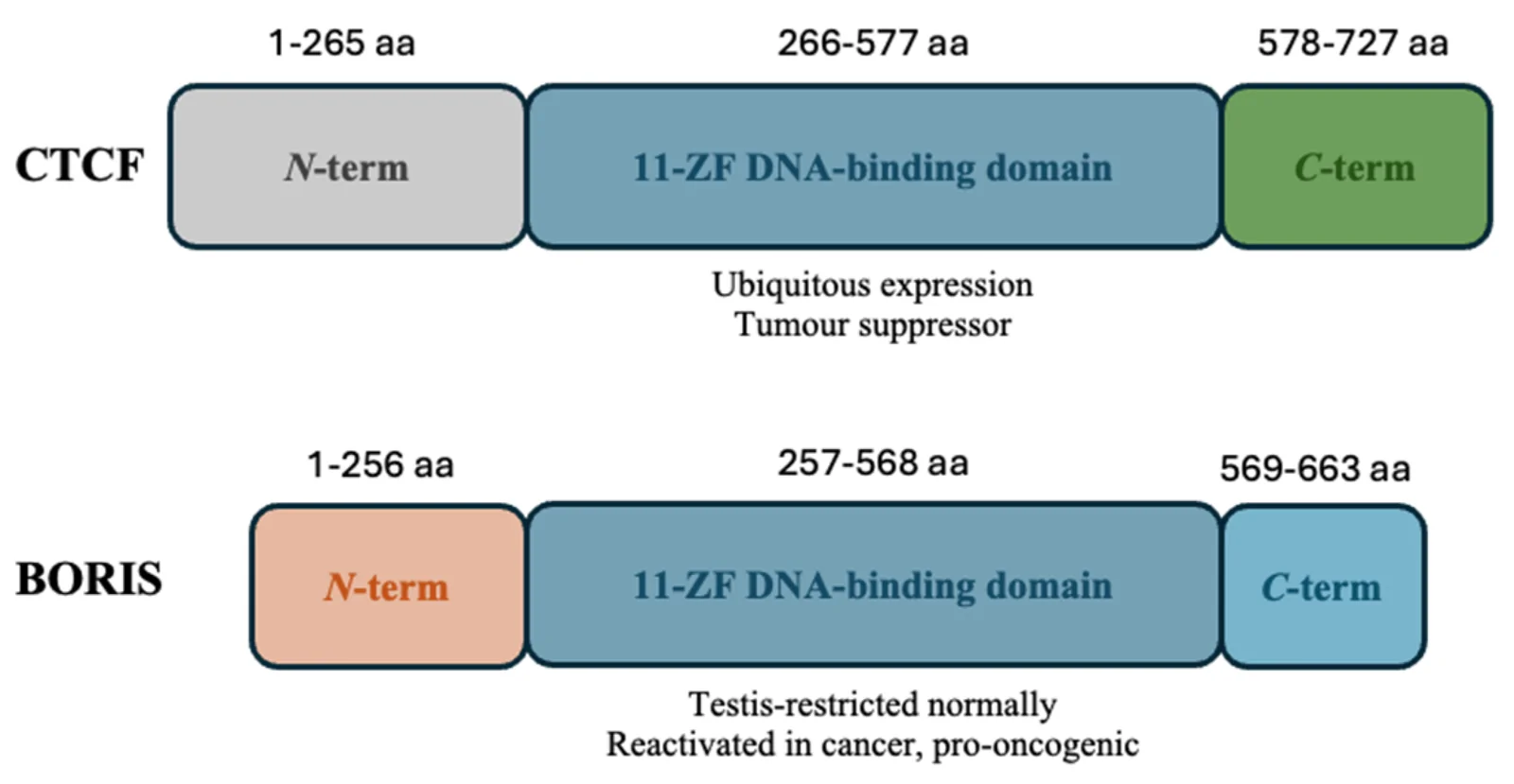
Domain structure of CTCF and BORIS. CTCF and BORIS share a highly conserved 11-zinc finger (ZF) DNA-binding domain, but different *N*- and *C*-terminal domains. Domain boundaries as per UniProt P49711 (CTCF_HUMAN) and Q8NI51 (CTCFL_HUMAN) [11].

BORIS is the paralogue of CTCF (CCCTC-Binding Factor), a ubiquitous protein with an essential role in transcriptional regulation, chromatin insulation and nuclear organisation. CTCF and BORIS share an almost identical DNA-binding domain that recognises the same DNA sequences [9]. However, due to structural divergence in their *N*- and *C*-terminal domains, BORIS and CTCF possess distinct developmental functions and expression patterns [10,11,12] (Figure 1). BORIS is expressed as at least 23 distinct isoforms grouped into six functional subfamilies (sf1–sf6), with variation in *N*-/*C*-terminal and zinc finger composition [10]. The domain structure shown in Figure 1 represents the canonical isoform.

In normal physiology, BORIS expression is restricted to the male germline, where it participates in epigenetic reprogramming during spermatogenesis, while CTCF is ubiquitously expressed across somatic tissues. In somatic cells, BORIS transcription is maintained in a silenced state through a set of repressive mechanisms acting across its three alternative promoters (A, B and C). While BORIS is transcribed from all three promoters in the normal testis, promoters A and C are preferentially utilised upon aberrant activation in cancer [12]. CpG methylation, p53-mediated transcriptional repression, and CTCF binding (which organises the surrounding chromatin into self-interacting topologically associating domains (TADs) via cohesin-mediated loops) collectively maintain BORIS silencing in somatic cells [12,13]. In cancer, this repression is disrupted by promoter hypomethylation, p53 mutation or loss, and CTCF mutation or haploinsufficiency, enabling aberrant BORIS reactivation across multiple cancer types. Once reactivated, BORIS occupies shared CTCF binding sites, displacing or co-occupying them with CTCF depending on site architecture, remodels chromatin architecture, and activates otherwise silenced gene programmes. These include oncogenes and germline-specific transcription networks that promote cell proliferation, invasion and therapy resistance [12,13,14].

This review examines the evidence for BORIS as a major contributor to epigenetic reprogramming and endocrine therapy resistance across prostate, breast, endometrial and ovarian cancers. We focus particularly on how aberrant BORIS activity interacts with hormone receptor signalling networks to facilitate transcriptional plasticity and therapeutic escape.

## 2. BORIS: Structure, Expression and Cancer Reactivation

The *BORIS* gene is located at chromosomal region 20q13.2, a locus subject to frequent amplification across multiple cancer types. The DNA-binding domains (DBDs) of *BORIS* and *CTCF* share approximately 74.1% amino acid identity, consistent with recognition of the same DNA sequences. In contrast, their *N*- and *C*-terminal domains show less than 15% identity, suggesting that interactions with distinct cofactors underlie their functional differences [15]. A critical region within the CTCF *N*-terminal domain is required for the cohesin-mediated extrusion of chromatin loops, a function that BORIS lacks [16]. By occupying shared binding sites, BORIS may weaken CTCF-dependent boundary function, which could alter TAD organisation and allow previously insulated enhancer-promoter connections to become aberrantly activated [13,16].

The *C*-terminal domain of BORIS confers a distinct set of protein–protein interaction and possesses multiple post-translational modification sites. CTCF can associate with histone acetyltransferase-containing complexes to function as a transcriptional activator, or bind to co-repressors and recruit histone deacetylases to function as a transcriptional repressor [17,18]. Unlike CTCF, BORIS lacks these repressive interactions. Instead, it recruits chromatin-opening machinery to the DNA binding sites it occupies. This divergence is evident at the hTERT telomerase promoter, where CTCF binding represses transcription in normal somatic cells, whereas BORIS occupancy at the same binding sites counteracts CTCF-mediated repression to activate hTERT transcription [19]. BORIS binding initiates the recruitment of the chromatin remodelling factor SRCAP, followed by the replacement of histone H2A with the histone variant H2A.Z. This results in a more relaxed chromatin state in the nucleosomes flanking CTCF binding sites, effectively overriding CTCF-mediated repression and activating alternative promoters [13].

However, BORIS does not function as a universal activator; its regulatory effects are genomic-context-dependent. At hominoid-specific SVA transposable elements, BORIS acts predominantly as a transcriptional repressor, cooperating with DNA and histone methylation machinery [20]. A similar context-dependent role has been observed in high-grade serous ovarian cancer, where BORIS binds the AR promoter and represses transcription [21]. These findings demonstrate that BORIS can function as either a transcriptional activator or repressor depending on the genomic locus and the surrounding chromatin landscape, interacting cofactors and cell-type specific regulatory networks.

## 3. Physiological Expression, Germline Function and Somatic Silencing

BORIS is normally expressed exclusively in male germ cells during spermatogenesis, where it participates in the epigenetic reprogramming of the paternal genome. The activation of BORIS is linked to the removal and re-establishment of methylation marks in early gametogenesis, with expression peaking in undifferentiated spermatogonia and post-meiotic spermatids [22]. Specifically, BORIS cooperates with the histone methyltransferase PRMT7 to facilitate de novo DNA methylation at the IGF2/H19 imprinting control region in male germ cells [23], whereas in somatic cells, these imprinting marks are protected by CTCF, which acts as an insulator on the maternal allele to prevent the expression of IGF2 [24].

Moreover, BORIS is a known inducer of multiple testis-specific genes that are suppressed by CTCF in somatic cells, including genes involved in meiosis and spermatogenesis, such as Gal3st1, SPANX-N and Prss50. Accordingly, BORIS-knockout male mice exhibit reduced testis size, defective sperm production and delayed spermatogenesis, highlighting its essential role in male germline development [25]. Although primarily a male germline factor, BORIS expression has also been detected in human oocyte, ovary, and various foetal tissues, suggesting an additional role in meiosis during oogenesis [10]. The germline-restricted expression of BORIS and the testis-specific gene networks it regulates form the biological foundation for its classification as a cancer-testis antigen (CTA), a group of genes that are normally restricted to male germ cells but are ectopically expressed in cancer [26]. The expression of cancer-testis genes correlates with carcinogenesis and chemotherapy resistance, making them reliable prognostic markers and attractive targets for immunotherapy [27].

**Figure 2:**
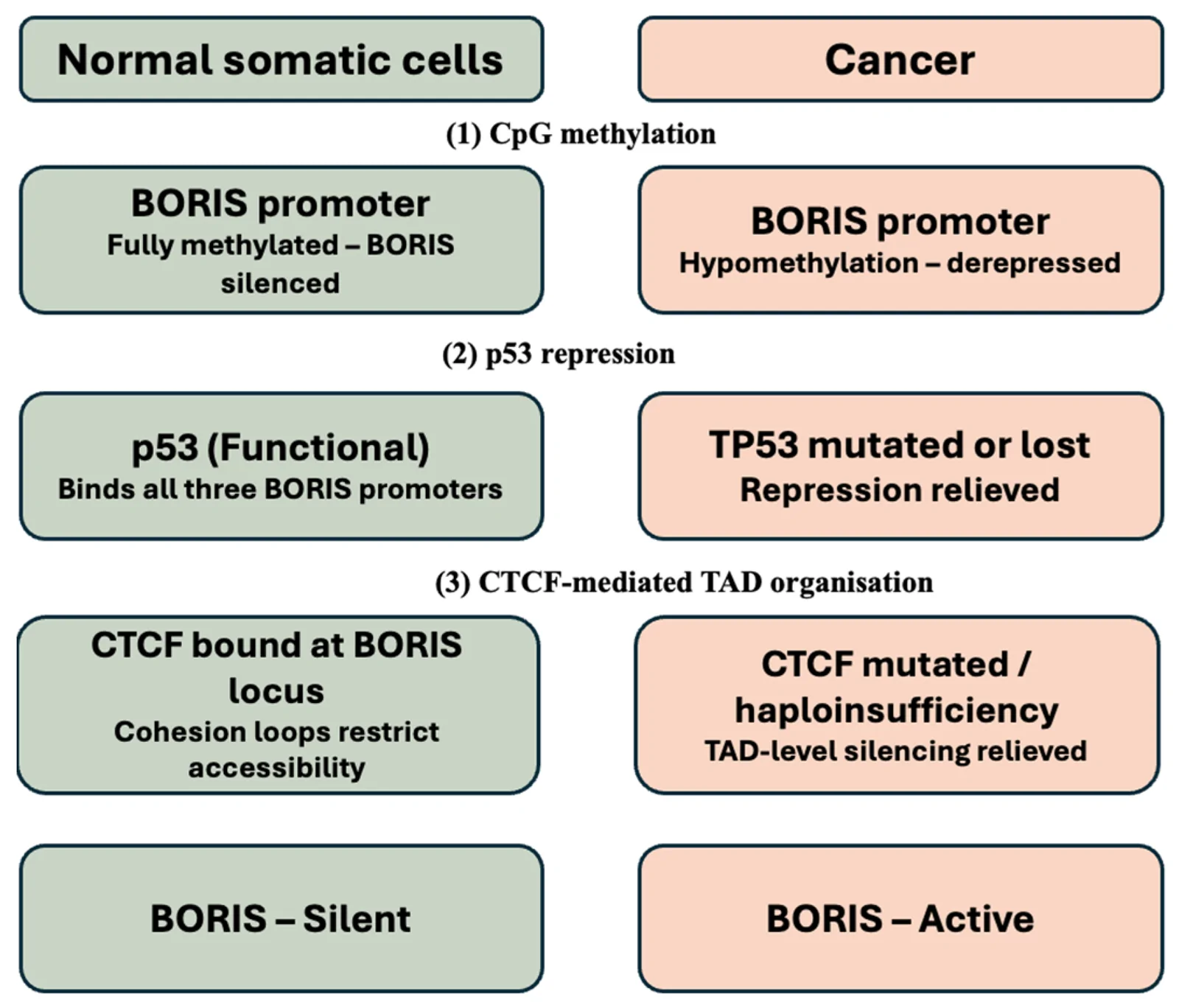
Mechanisms of BORIS reactivation in cancer. In normal somatic cells, BORIS expression is maintained in a silenced state through three repressive mechanisms: CpG methylation, p53-mediated transcriptional repression, and CTCF-mediated topologically associating domain (TAD) organisation. In cancer, CpG hypomethylation of the BORIS promoter, TP53 mutation or loss, and CTCF mutation or haploinsufficiency relieve these repressive constraints, which may result in BORIS activation.

As previously discussed, in somatic cells, BORIS expression is silenced through several repressive mechanisms operating across its alternative promoters, including CpG methylation, p53-mediated repression, and CTCF-mediated TAD organisation [12,13]. In cancer, these regulatory mechanisms are disrupted as a consequence of BORIS promoter demethylation, p53 mutation/loss, and somatic mutation or haploinsufficiency of CTCF, which occurs most frequently in endometrial cancer [28]. This may further relieve CTCF-mediated, TAD-level silencing. The co-occurrence of these events in advanced hormone-driven cancers can create a permissive epigenetic environment for BORIS reactivation (Figure 2). We hypothesise that BORIS reactivation coincides with the stage at which endocrine resistance emerges, and that BORIS may be both a product of and an active contributor to the epigenetic reprogramming that underlies therapy resistance. Evidence that BORIS can reshape enhancer-promoter architecture in an established therapy-resistance setting (neuroblastoma) is discussed in Section 4.1 [14].

## 4. Mechanisms of BORIS-Driven Transcriptional Reprogramming

The primary studies reporting BORIS expression and functional evidence across hormone-driven cancers are summarised in Table 1.

### 4.1. BORIS and CTCF at Shared Binding Sites and Chromatin Boundaries

It has been proposed that BORIS competes with CTCF for DNA binding, leading to the complete replacement of CTCF at shared target sites and the consequent disruption of CTCF-mediated chromatin organization and gene regulation. However, genome-wide studies have demonstrated that the relationship between the two proteins is more complex, with both exclusive and shared occupancy determined by the architecture of the binding site. Comparative ChIP-seq analyses have established that CTCF binding sites are not homogeneous but can be subdivided into two functionally distinct classes. Single CTCF target sites (1xCTSs) comprise one CTCF motif and are bound by CTCF alone. Conversely, clustered sites (2xCTSs) contain two or more clustered CTCF motifs and are simultaneously co-occupied by both CTCF and BORIS in BORIS-expressing germ and cancer cells, or by BORIS alone. Notably, co-occupied 2xCTSs exhibit significantly stronger CTCF and BORIS ChIP-seq signals than CTCF-only or BORIS-only bound sites, supporting a model of cooperative rather than simple competitive binding. Since BORIS lacks the *N*-terminal sequence essential for cohesin recruitment, its occupancy at shared binding sites cannot support the cohesin-dependent function that underlies CTCF-mediated chromatin insulation. Therefore, the replacement of CTCF by BORIS at these sites may result in TAD boundary disruption [42].

Disruption of TAD boundaries can rewire enhancer-promoter contacts and cause disease by allowing genes to interact with regulatory elements that are normally insulated. In cancer, TAD boundary disruption arises through genomic rearrangements or mutations affecting boundary elements [43]. In contrast, BORIS is proposed to disrupt chromatin boundary function through a non-mutational mechanism involving altered CTCF occupancy rather than sequence deletion, making it potentially reversible and therapeutically targetable in cancer [42].

Taken together, these findings indicate that CTCF displacement describes only one of BORIS’s modes of action. At single CTCF sites, displacement or replacement is plausible. At clustered 2xCTSs, BORIS and CTCF co-occupy the DNA and show stronger binding than either alone [42]. In FTSECs, BORIS expression increases CTCF occupancy at hundreds of loci [30]. In neuroblastoma, BORIS occupancy rises while CTCF binding is unchanged [14]. Which mode predominates probably depends on cell type, site architecture and chromatin state.

Evidence that BORIS can reshape regulatory chromatin architecture in a therapy-resistance setting comes from neuroblastoma. In ALK-mutated, MYCN-amplified neuroblastoma cells, resistance to ALK inhibition arose through the loss of MYCN expression followed by BORIS overexpression [14]. Most BORIS peaks specific to resistant cells co-localised with CTCF binding sites, while most CTCF peaks were shared between sensitive and resistant cells, indicating that BORIS occupied CTCF sites without a global loss of CTCF binding. BORIS-dependent chromatin interactions, mapped by SMC1A HiChIP, were linked to newly formed super-enhancers, which drove ectopic expression of a subset of proneural transcription factors. BORIS knockdown partly restored sensitivity to ALK inhibition [14]. Together, these findings provide mechanistic evidence that BORIS can act alongside CTCF at shared sites to rewire enhancer-promoter interactions during therapy resistance.

### 4.2. Reprogramming of CTCF Binding Sites into Alternative Transcriptional Start Sites

**Figure 3:**
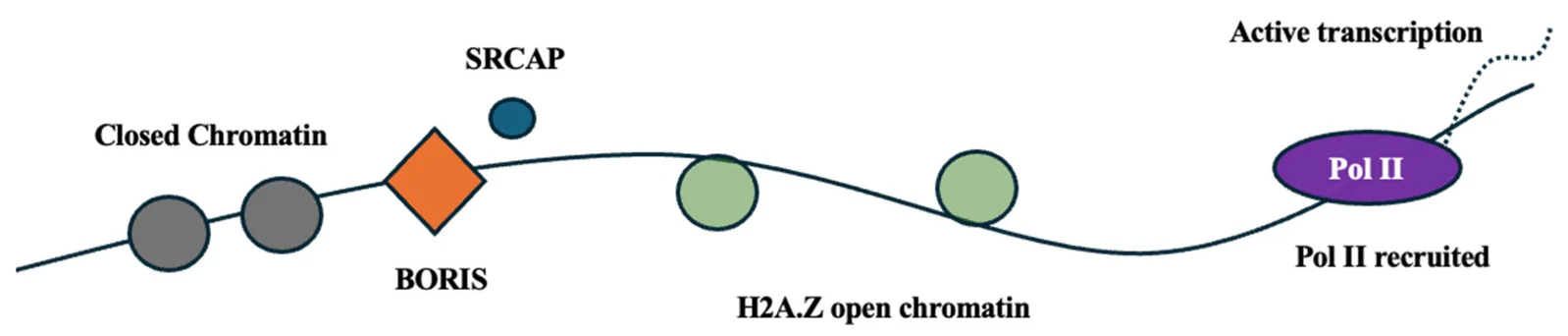
BORIS-mediated chromatin remodelling at clustered CTCF binding sites. BORIS binding at a clustered CTCF site (2xCTS) recruits the chromatin-remodelling factor SRCAP through a direct protein–protein interaction. SRCAP promotes the exchange of canonical histone H2A for the histone variant H2A.Z, generating a more accessible chromatin environment. This facilitates recruitment of RNA polymerase II (Pol II) and additional transcription factors, activating transcription initiation.

One of the key mechanisms through which BORIS promotes oncogenic transcription is by converting transcriptionally inactive CTCF binding sites into de novo promoters, initiating aberrant transcription from genomic regions that are normally silent in somatic cells. Upon BORIS expression, intergenic and intronic CTCF binding sites become epigenetically reprogrammed into active transcriptional start sites, recapitulating a germline-specific transcriptional program in cancer cells [13]. BORIS binding at clustered CTCF binding sites recruits the chromatin-remodelling factor SRCAP through a direct protein–protein interaction. SRCAP recruitment promotes the exchange of canonical histone H2A for the histone variant H2A.Z in nucleosomes flanking the binding site, generating a more accessible chromatin environment that facilitates the subsequent recruitment of RNA polymerase II and additional transcription factors. Consequently, previously inactive CTCF-bound sites acquire promoter activity and gain histone and post-translational modifications associated with active transcription, including H2A.Z, H3K4Me3, H3K27Ac and CAGE-seq signals (which measure transcription start site activity), indicative of active transcription initiation [13] (Figure 3).

The transcriptional outputs of BORIS-mediated chromatin reprogramming are extensive. BORIS activates the expression of CTAs, long non-coding RNAs, pseudogenes and transposable elements [13]. BORIS also activates the expression of hTERT by binding the first exon of the gene in embryonic and ovarian cancer cells, thereby promoting a stem-like state [19,32]. In normal somatic cells, CTCF binds this site to repress hTERT. In the embryonic and ovarian cancer studies, BORIS and CTCF are co-expressed and both bind the first exon in vivo; BORIS counteracts CTCF-mediated repression, possibly by competing with or substituting for CTCF at this site [19]. The CTCF binding sites reprogrammed by BORIS, and the transcriptional networks that are subsequently activated, are determined by the chromatin landscape and cell-type-specific regulatory environment of the tumour cell [42].

### 4.3. BORIS and Hormone Receptor Regulation

The chromatin remodelling mechanisms outlined previously influence hormone receptor signalling through both direct and indirect mechanisms, with effects that vary according to tumour type and disease stage. Indirectly, CTCF plays a role in maintaining the chromatin architecture that governs hormone receptor transcriptional activity. Directly, BORIS was shown to bind hormone receptor gene promoters and modulate their transcription. In high-grade serous ovarian cancer, genome-wide studies of BORIS knockout identified AR as the major transcription factor within the BORIS-associated regulatory network, with ChIP showing that BORIS is a direct transcriptional repressor of AR at its promoter [21]. This repression extends downstream to established AR targets including FN1, FAM129A and CD97, which are linked to chemoresistance, metastasis and poor prognosis in ovarian cancer [21]. Beyond AR, a subsequent analysis of OVCAR3 transcriptomes identified PI3K-Akt signalling as a second major pathway, alongside processes linked to cell motility and extracellular matrix organisation, with FGFR1, VEGFA, WNT9A and FZD4 emerging as additional BORIS-associated genes [31].

Indirectly, BORIS is proposed to mediate hormone receptor signalling by altering CTCF occupancy at shared binding sites. In ER-positive breast cancer, reduced CTCF occupancy at chromatin compartment boundaries is a consistent feature of both active ER signalling and endocrine-resistant states. Oestradiol-induced dynamic compartments show enhanced ERα binding alongside decreased CTCF binding. In tamoxifen-resistant breast cancer cells, 3D chromatin interactions change extensively at ER binding sites and CTCF-bound anchors, in association with altered ER-regulated gene expression [44,45].

**Figure 4:**
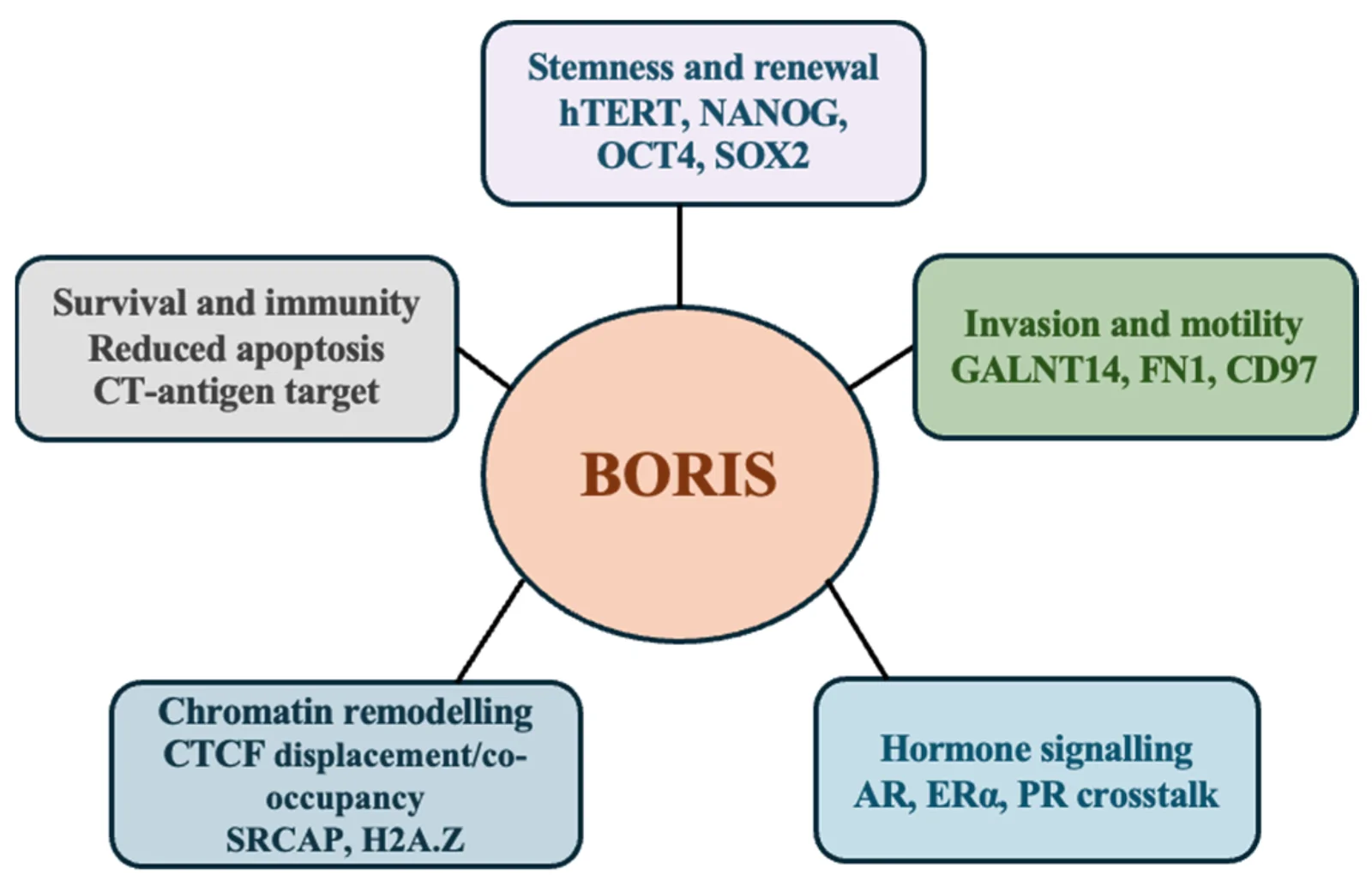
Functional consequences of BORIS expression in cancer. BORIS drives a range of pro-tumorigenic phenotypes through downstream programs: induction of stemness and self-renewal markers, increased invasion and motility, crosstalk with hormone receptor signalling, chromatin remodeling at target loci through CTCF displacement or co-occupancy and SRCAP/H2A.Z-mediated nucleosome exchange and effects on cell survival and immune recognition, including reduced apoptosis. These functions position it as a cancer-testis antigen vaccine target.

Collectively, these mechanisms give rise to a number of downstream functional programmes and phenotypic alterations, including the induction of stemness and self-renewal, enhanced invasion and motility, hormone receptor crosstalk, chromatin remodelling at target loci and altered survival/immune recognition, several of which recur across the hormone-driven cancers discussed in the following section (Figure 4).

## 5. BORIS in Hormone-Driven Cancers: Disease-Specific Evidence

### 5.1. Ovarian Cancer

Ovarian cancer represents a heterogeneous group of malignancies with distinct histological subtypes, molecular profiles and hormonal dependencies. This section focuses on high-grade serous carcinoma (HGSC), which accounts for the majority of ovarian cancer deaths and is believed to originate from fallopian tube secretory epithelial cells (FTSECs) [46]. HGSC is characterised by *TP53* mutation in 96% of cases [47], widespread genomic instability, and global DNA hypomethylation [48], which represents the precise epigenetic state that constitutes a permissive mechanism for the BORIS reactivation (discussed in Section 3).

BORIS is expressed in the majority of epithelial ovarian cancer cases, and its reactivation is associated with both promoter-specific and global DNA hypomethylation. Elevated BORIS expression correlates significantly with advanced tumour stage and poor prognosis [29,30]. Notably, the BORIS isoform profile in ovarian cancer differs from that observed in germline tissues. In hypomethylated ovarian tumours, the sf1 isoform subfamily, which is undetectable in normal ovarian tissues, is activated together with sf2, and the ratio of sf2 and sf6 expression to CTCF is elevated. These findings suggest that BORIS reactivation in ovarian cancer is driven by isoform-specific promoter demethylation rather than uniform activation of the entire locus [49]. The tumour-restricted expression of the sf1 isoform, together with its absence in normal tissues, highlights its potential as a biomarker for minimally invasive biopsy applications.

The functional consequences of BORIS expression in HGSC precursor cells were directly characterised by Hillman et al. [30] who expressed BORIS in FTSECs and examined the resulting downstream transcriptional and chromatin changes. BORIS expression significantly enhanced cell motility and invasion, demonstrating that BORIS expression in ovarian cancer precursor cells is sufficient to confer a pro-invasive phenotype. Transcriptomic analysis revealed alterations in cancer-associated gene expression networks, including fatty acid metabolism, TNF signalling, cell migration and ECM-receptor interactions [30]. The key downstream mediator of BORIS-driven invasiveness was identified as *GALNT14*, a glycosyltransferase gene implicated in cancer migration and invasion. BORIS induced *GALNT14* expression, while *GALNT14* knockdown significantly attenuated BORIS-induced cell motility and invasion [30]. At the chromatin level, ChIP-seq analysis revealed that BORIS expression in FTSECs was associated with de novo and enhanced CTCF occupancy at hundreds of genomic loci, many of which corresponded to transcriptionally activated target genes, including *GALNT14*, indicating that BORIS remodels the CTCF cistrome in ovarian cancer precursor cells rather than simply displacing CTCF at pre-existing sites [30].

One of the most significant mechanistic insights into BORIS function in ovarian cancer is its regulation of AR signalling through binding to the *AR* promoter. Genome-wide knockout and knockdown experiments in the OVCAR3 cell line identified 130 differentially expressed BORIS target genes. Network enrichment analysis revealed AR as one of the main regulators of this BORIS-associated regulatory network. *BORIS* knockdown led to increased *AR* expression alongside upregulation of *FN1*, *FAM129A* and *CD97*, genes associated with chemoresistance and metastasis in ovarian cancer. This demonstrates that BORIS actively suppresses AR and its downstream target genes involved in metastasis [21]. Analysis of public expression data from 343 ovarian cancer patient samples supported the clinical relevance of this regulatory axis, as the BORIS target gene expression patterns observed in vitro were recapitulated in patient tumour data. This finding establishes a direct mechanistic link between BORIS activity and hormonal signalling dysregulation in ovarian cancer [21].

Beyond invasion and AR regulation, BORIS expression correlates with stemness factors (NANOG, OCT4 and SOX2), alongside the cancer stem cell marker CD44 and hTERT [19,32]. This suggests that BORIS-expressing cells are enriched for stem-like properties that may contribute to chemotherapy resistance and disease recurrence [32]. BORIS-driven hTERT activation in ovarian cancer cells further links BORIS activity to telomerase reactivation and cellular immortalisation [19]. Together, these findings suggest that BORIS acts as a multifunctional epigenetic regulator in ovarian cancer that promotes invasion, suppresses the AR regulatory network, activates stemness transcriptional programs and sustains cellular immortalisation through hTERT, collectively contributing to therapy resistance.

### 5.2. Breast Cancer

Breast cancer is the most commonly diagnosed female malignancy worldwide, with approximately 70–80% of cases expressing oestrogen receptor alpha (ERα) and/or progesterone receptor (PR), classifying them as hormone-dependent cancers [50]. BORIS expression was detected in >70% of breast tumour samples examined by immunohistochemical staining, with high BORIS expression correlating significantly with increased ERα and PR expression [34]. The link between BORIS and hormone receptor status was further evidenced by the ability of BORIS to induce *PR* gene expression in breast cancer cells, suggesting that BORIS enhances hormone receptor-dependent transcriptional programmes in this context [34]. Additionally, BORIS protein was detected in the leukocytes of breast cancer patients but not in healthy controls, suggesting a potential role for BORIS as an early detection biomarker in peripheral blood [33].

The functional consequences of BORIS in breast cancer cells are subtype dependent. In invasive breast cancer cells, *BORIS* silencing reduced the expression of hTERT, the pluripotency transcription factors NANOG, OCT4, SOX2, and BMI1, and the cancer stem cell markers ABCG2, CD44, and ALDH1. This was accompanied by a reduced sphere formation capacity, indicating that BORIS sustains a cancer stem cell programme in invasive disease [36]. In contrast, in the non-invasive, ER-positive MCF-7 cell line, *BORIS* silencing induced an EMT gene signature, the acquisition of a CD44+/CD24− cancer stem cell phenotype, increased hTERT expression and enhanced cell survival. This demonstrates that BORIS constrains, rather than promotes, stemness in non-invasive ER-positive breast cancer cells [36]. Furthermore, siRNA-mediated knockdown of *BORIS* induced selective apoptosis in breast cancer cells while leaving normal breast epithelial cells unaffected, establishing BORIS as a functional survival factor and highlighting its potential as a therapeutic target [35].

Together, these findings suggest that BORIS acts as an epigenetic regulator in the majority of hormone receptor-positive breast tumours, where its expression positively correlates with ERα and PR levels and sustains cancer stem cell programmes in invasive disease. Notably, direct experimental evidence linking BORIS to endocrine therapy resistance in breast cancer remains absent, representing a critical and tractable gap that warrants investigation.

### 5.3. Endometrial Cancer

Endometrial cancer is a malignant tumour originating from the endometrial epithelium. The majority of cases are classified as Type I endometrioid carcinoma, a hormone-driven subtype in which sustained oestrogen stimulation drives proliferation through ERα and PR signalling [51,52]. BORIS expression in uterine cancers was first systematically characterized by Risinger et al. (2007), who identified a high incidence of BORIS expression across a panel of uterine cancer samples, establishing endometrial cancer as a tumour type in which BORIS reactivation is a consistent feature [38].

The most comprehensive characterisation of BORIS in endometrial cancer was provided by Hoivik et al. [39], who examined BORIS expression and promoter methylation across precursor lesions, primary tumours, and metastatic samples. Bisulphite sequencing revealed a stepwise loss of *BORIS* promoter methylation from precursor lesions through primary tumours to metastatic disease. This loss of methylation inversely correlated with *BORIS* mRNA expression across disease stages, indicating that *BORIS* promoter demethylation increases progressively as disease advances [39]. High BORIS expression correlated significantly with non-endometrioid histology, high tumour grade, and reduced recurrence-free and overall survival in both the primary cohort and TCGA validation data, a pattern consistent with BORIS functioning as an epi-driver gene in endometrial cancer [39].

High BORIS expression in primary endometrial tumours was significantly associated with downregulation of *ESR1*, *PGR*, and *AR* mRNA, demonstrating an inverse relationship between BORIS and hormone receptor gene expression [39]. This pattern is consistent with the repressive role of BORIS observed at the *AR* promoter in ovarian cancer [21]. The loss of PR expression is a recognised mechanism through which endometrial tumours escape progesterone-mediated growth suppression and develop resistance to progestin therapy. The inverse correlation between BORIS and PR expression observed by Hoivik et al. [39] raises the possibility that BORIS contributes to this process by epigenetically silencing *PR*. This hypothesis has not yet been directly tested in endometrial cancer cells and represents a clear priority for future experimental work.

### 5.4. Prostate Cancer

Prostate cancer is the most frequently diagnosed malignancy in men and a leading cause of cancer-related death worldwide. Disease progression is characterised by an initial dependence on AR signalling, followed by the emergence of castration-resistant disease [53]. The most comprehensive primary evidence for BORIS in prostate cancer was provided by Cheema et al. [40], who examined BORIS expression across a panel of prostate cancer patient tissue samples. BORIS protein was detected in prostate cancer tissue but absent in matched benign prostatic epithelium, confirming its cancer specificity. Immunohistochemical analysis revealed a significant positive correlation between BORIS expression and both Gleason score and pathological tumour stage, directly linking BORIS levels to tumour grade, invasiveness, and metastatic potential [40]. These observations establish BORIS as a cancer-specific marker in prostate cancer, suggesting that it may contribute to the aggressive phenotype of high-grade disease, though the functional mechanisms underlying this association have not yet been directly investigated in prostate cancer cell models.

Hoffmann et al. [41] examined BORIS expression in prostate and bladder carcinoma cell lines and tissue samples. In most prostate carcinoma cell lines, BORIS expression was not significantly elevated compared to normal prostate tissue. However, a subset of individual prostate tumour samples showed modestly increased BORIS expression accompanied by promoter hypomethylation, consistent with the methylation-dependent reactivation mechanism [41]. To test whether DNA methylation was responsible for maintaining BORIS silencing, prostate cancer cell lines were treated with 5-aza-2′-deoxycytidine, a DNA demethylating agent that removes methyl groups from CpG sites and restores expression of silenced genes. This treatment dramatically induced BORIS expression, indicating that DNA methylation plays a key role in maintaining *BORIS* silencing in prostate epithelial cells and that demethylating agents can reactivate BORIS expression [41].

## 6. Clinical and Translational Implications

### 6.1. BORIS as a Biomarker in Hormone-Driven Cancers

BORIS holds a strong potential as a clinical biomarker in hormone-driven cancers because its utility extends beyond simply indicating abnormal epigenetic regulation. It actively modulates hormone receptor signalling and may predict patient responses to endocrine therapy, making it particularly relevant in cancers that are hormone-dependent.

Crucially, the inverse relationship between BORIS and hormone receptor expression has now been reported in two of the four cancers discussed in this review. In endometrial cancer, high BORIS levels are associated with lower expression of ERα, PR and AR in primary tumours [39]. Similarly, in ovarian cancer, BORIS knockdown upregulates AR expression [21]. These findings suggest that rather than acting merely as a generic marker of aggressive disease, BORIS could serve as a specific indicator of hormone receptor loss, a hallmark of endocrine therapy resistance across prostate, breast, endometrial, and ovarian cancers. If confirmed prospectively, elevated BORIS expression at diagnosis or pre-treatment could help identify patients unlikely to benefit from endocrine therapy, thereby guiding personalised treatment decisions earlier in the clinical timeline.

As a prognostic biomarker, the most robust evidence emerges from ovarian and endometrial cancers. In ovarian cancer, a high BORIS/CTCF expression ratio correlates significantly with advanced-stage disease, as well as reduced progression-free and overall survival [30]. Utilising the BORIS/CTCF ratio, rather than evaluating BORIS expression in isolation, is mechanistically meaningful in this context because CTCF normally maintains three-dimensional chromatin organisation and regulates hormone receptor gene expression. When BORIS levels are elevated relative to CTCF, this balance is disrupted, potentially altering hormone receptor-regulated loci and driving tumour progression. Consequently, the BORIS/CTCF ratio may provide a superior, more informative prognostic indicator. In endometrial cancer, high BORIS expression is linked to non-endometrioid histology, higher tumour grade, and poorer recurrence-free and overall survival in both a primary patient cohort and TCGA datasets [39]. Additionally, loss of methylation at the *BORIS* promoter was observed from precursor lesions through primary tumours to metastatic lesions, suggesting that *BORIS* promoter methylation could be used as a molecular tracking tool for disease progression. Because DNA methylation can be mapped in circulating cell-free DNA (cfDNA) using established bisulphite sequencing methods [54], *BORIS* promoter methylation status has potential as a non-invasive liquid biopsy marker. However, whether cfDNA-based *BORIS* promoter methylation status can reliably predict clinical progression or the onset of endocrine resistance remains an open question that warrants future research.

In breast cancer, BORIS has been detected in the peripheral blood leukocytes of patients but remains undetectable in non-cancer controls [33]. Similarly, in prostate cancer, BORIS expression is strictly cancer-specific relative to benign prostatic tissue and correlates with both Gleason score and tumour stage [40]. Together, these findings support a possible diagnostic and prognostic role for BORIS across these malignancies, though large-scale prospective validation is required to validate its clinical utility.

### 6.2. BORIS as a Cancer-Testis Antigen Immunotherapy Target

The cancer-testis antigen (CTA) characteristics of BORIS make it an attractive target for cancer immunotherapy. Under physiological conditions, BORIS expression is restricted to germ cells and is completely absent from normal somatic tissues. However, it is abnormally re-expressed in multiple malignancies, including breast, ovarian, prostate and endometrial cancers. Due to this strict tumour-restricted expression profile, BORIS was included in the National Cancer Institute’s priority list of cancer vaccine target antigens, which highlights the most promising candidates for clinical translation [55].

Preclinical evidence for BORIS-directed immunotherapy has been generated primarily in breast cancer models. A therapeutic vaccine expressing a modified BORIS protein that lacks the DNA-binding domain demonstrated potent anti-tumour activity in both the aggressive 4T1 murine breast cancer model and the 13,762 rat mammary carcinoma model. Vaccine administration significantly reduced tumour growth, displaying complete regression in approximately 50% of the treated animals. Re-challenge experiments also demonstrated long-lasting immune memory, indicating that the vaccine confers sustained protection against tumour recurrence [37]. Remarkably, the vaccine remained highly effective despite BORIS expression being restricted to only a small subpopulation of cancer stem cells (CSCs) within the bulk tumour [37]. This aligns with data showing that BORIS silencing in cancer stem cells triggers senescence and cell death [56], suggesting that the selective immunological elimination of BORIS-expressing CSCs is sufficient to suppress overall tumour growth.

A systematic review and meta-analysis of 73 CTA vaccine trials reported a pooled objective response rate of 13% and a disease control rate of 39%, with superior outcomes observed using intravenous delivery and dendritic cell-based vaccine platforms [57]. While these benchmark studies confirm that CTA vaccines can elicit objective clinical responses, optimising vaccine design, adjuvant selection and combination strategies is still required.

Despite these encouraging preclinical findings, BORIS-targeted vaccines have not yet transitioned into clinical trials for hormone-driven cancers. A major hurdle is the architectural complexity of BORIS itself, which encodes at least 23 distinct splice variants with highly heterogeneous expression profiles across different cancer types [10]. Consequently, next-generation vaccine designs must specifically target conserved antigenic regions shared across clinically relevant oncogenic BORIS isoforms, while strictly avoiding cross-reactivity with physiological germline expression in spermatocytes.

### 6.3. Targeting the BORIS-Dependent Chromatin Programme

Beyond immunotherapy, the mechanistic evidence reviewed in Section 3 and Section 4 identifies the BORIS-dependent chromatin programme as a rational therapeutic target in endocrine-resistant hormone-driven cancers. Since no direct BORIS inhibitor is currently available, targeting the downstream chromatin regulators and signalling pathways controlled by BORIS may provide an alternative strategy.

One promising target is the SRCAP/H2A.Z chromatin-remodelling pathway. BORIS recruits the SRCAP complex to clustered CTCF-binding sites, leading to the incorporation of the histone variant H2A.Z and creating an open chromatin environment that promotes alternative transcription initiation [13]. Blocking SRCAP activity could therefore interfere with these BORIS-driven chromatin alterations. However, no pharmacological agents specifically targeting SRCAP are available, currently limiting this approach. Alternatively, BORIS-regulated regions are enriched for active histone marks such as H3K27Ac and H3K4Me3 [13], making them potential targets for BET bromodomain inhibitors. These small molecules inhibit BRD4 binding to acetylated histones, thereby disrupting transcriptional elongation at active enhancers and oncogenic promoters. In castrate-resistant prostate cancer (CRPC), the dual inhibition of EZH2 and BET proteins has shown encouraging preclinical efficacy by suppressing c-MYC and NF-kB signalling, two oncogenic drivers frequently upregulated in CRPC and associated with aberrant epigenetic reprogramming [58]. Given that BORIS-driven chromatin remodelling similarly activates broad oncogenic transcriptional networks, this combinatorial approach may be highly relevant in BORIS-expressing, endocrine-resistant tumours, although a direct mechanistic link between BORIS and c-MYC or NF-kB activation in prostate cancer remains to be validated experimentally.

DNA demethylating agents such as 5-azacitidine and decitabine are also relevant in this context. Treatment with 5-aza-2′-deoxycytidine markedly reactivates BORIS expression in prostate cancer cell lines [41]. This raises a critical safety consideration for their use alongside endocrine therapies since these agents might inadvertently reactivate BORIS and accelerate treatment resistance, a possibility that warrants thorough preclinical investigation.

BORIS activity in ovarian cancer has been linked to the PI3K-Akt signalling pathway. BORIS regulates PI3K-Akt signalling in ovarian cancer cells, establishing it as a critical mediator of tumour progression and a candidate target for personalised therapy [31]. This finding is clinically significant because PI3K-Akt pathway inhibitors are already approved in several oncology settings, providing a strong rationale for combining them with endocrine therapy in BORIS-overexpressing ovarian cancer.

## 7. Conclusions and Future Directions

This review has examined the evidence for BORIS as an epigenetic driver of transcriptional reprogramming and endocrine therapy resistance across four hormone-driven malignancies: prostate, breast, endometrial and ovarian cancers. Across these diverse disease contexts, a remarkably consistent pattern emerges: BORIS is aberrantly reactivated in cancer through the loss of CpG methylation, p53 dysfunction and the disruption of CTCF-mediated chromatin organisation. Once expressed, BORIS occupies shared CTCF binding sites, displacing or co-occupying them with CTCF, reprograms clustered sites into alternative transcriptional start sites and activates germline gene networks that sustain proliferation, stemness, and cell survival. The mechanistic consequence of this reprogramming is not simply the activation of individual oncogenes, but a broader reorganisation of chromatin architecture that disrupts the three-dimensional regulatory networks governing hormone receptor transcriptional programmes. This solidifies the importance of BORIS as a central epigenetic driver of endocrine therapy resistance.

The disease-specific data reviewed here reveal both shared and divergent roles for BORIS across hormone-driven cancers. In ovarian cancer, BORIS directly represses AR regulatory network and promotes invasiveness through GALNT14, with expression levels correlating with advanced stage and poor survival. In breast cancer, BORIS expression positively correlates with ERα and PR levels and sustains cancer stem cell programmes in invasive disease, though its relationship with stemness is reversed in non-invasive ER-positive cells, highlighting the importance of tumour subtype context in interpreting BORIS function. In endometrial cancer, high BORIS expression inversely correlates with ERα, PR and AR gene expression, supporting the hypothesis that BORIS actively promotes hormone receptor loss and progesterone resistance, the central driver of treatment failure in this disease. In prostate cancer, BORIS expression correlates with Gleason score and clinical stage, and demethylating agents used in combination with AR-directed therapies can reactivate BORIS expression, with unknown consequences for resistance progression.

While this review highlights the emerging role of BORIS across hormone-driven cancers, several fundamental questions remain unresolved. Most notably, no study has directly tested whether BORIS functionally modulates ERα or PR signalling in breast or endometrial cancer cells. The current literature establishes correlative expression patterns but lacks direct functional mechanisms, highlighting a key priority for future research. It is also unclear whether BORIS reactivation occurs as an early event in the development of endocrine resistance or emerges later as a consequence of prolonged epigenetic adaptation. Resolving this timeline will require longitudinal tracking of BORIS expression and chromatin occupancy in cell line models and patient material. Furthermore, the contribution of individual BORIS isoforms to malignant behaviour has been detailed exclusively in ovarian cancer; whether specific isoforms drive distinct gene expression programmes in breast, endometrial, and prostate cancers remains unknown. Additionally, whether BORIS promoter methylation can be captured in blood-based cfDNA and deployed as a non-invasive marker of endocrine resistance has not been tested in any of the four cancer types reviewed here, despite being technically feasible with current methods. Finally, the promising anti-tumour efficacy of BORIS-targeting vaccines in rodent breast cancer models has yet to be translated into human hormone-driven settings. Overcoming the hurdle of isoform specificity to ensure that an elicited immune response targets the correct, oncogenic BORIS isoforms expressed in tumour cells, while sparing physiological germline cells, remains the ultimate goal to address before clinical translation can succeed.

## Figures and Tables

**Table 1:** Summary of published studies reporting BORIS expression and functional evidence across hormone-driven cancers.

Cancer Type	Ref.	Study Type	Model	BORIS Expression Finding	Key Functional Evidence
**Ovarian**	[29]	Clinical	75 EOC samples	BORIS/CTCF ratio associated with advanced stage and reduced OS/PFS	BORIS promoter hypomethylation correlates with global hypomethylation and advanced disease
**Ovarian**	[30]	Functional/ ChIP-seq	FTSECs (HGSC precursor)	BORIS expressed in majority of EOCs; correlates with advanced stage and poor prognosis	BORIS increases motility and invasion; GALNT14 identified as downstream effector
**Ovarian**	[21]	KO/KD+patient data	OVCAR3; 343 patient samples	BORIS controls AR regulatory network; AR is major transcription factor hub in BORIS-associated network	BORIS KD increases AR, FN1, FAM129A, CD97 expression; pattern validated in patient tumour data
**Ovarian**	[31]	Systems biology	Ovarian cancer cell lines	BORIS regulates PI3K-Akt signalling network	PI3K-Akt identified as targetable downstream pathway; basis for personalised therapy
**Ovarian**	[32]	Functional	OVCAR3; NTERA-2,	BORIS-positive cells express high hTERT, NANOG, OCT4, SOX2, CD44 levels	BORIS silencing reduces hTERT and stemness markers; links BORIS to cancer stem cell phenotype
**Breast**	[33]	Clinical	Breast cancer patient leukocytes	BORIS protein detected in peripheral blood leukocytes of patients; low in non-cancer control groups	Potential as early detection blood biomarker
**Breast**	[34]	Clinical	58 breast tumour samples	BORIS detected in 70.7% of breast tumours; positively correlates with ERα ± high PR levels	BORIS amplifies hormone receptor-dependent transcription
**Breast**	[35]	Functional/ siRNA	Breast cancer cell lines	BORIS expressed in breast cancer cell lines	siRNA-mediated BORIS KD induces selective apoptosis in cancer cells
**Breast**	[36]	Functional	MDA-MB-231; MCF7	BORIS expressed in invasive and non-invasive breast cancer lines	In invasive cells: BORIS KD reduces hTERT, NANOG, OCT4, ABCG2, CD44, ALDH1 and sphere formation. In MCF7: BORIS KD induces EMT
**Breast**	[37]	Preclinical vaccine	4T1 mouse; 13,762 rat models	BORIS restricted to CSC subpopulation within tumour	VRP-mBORIS vaccine suppresses tumour growth; complete regression in ~50% animals
**Endometrial**	[38]	Expression profiling	Uterine cancer samples	High incidence of BORIS expression across uterine cancer panel	First systematic characterisation of BORIS as a recurrent CTA in endometrial cancer
**Endometrial**	[39]	Clinical	Precursor lesions, primary tumours, metastases; TCGA validation	High BORIS expression correlates with non-endometrioid histology, high grade, reduced RFS and OS; stepwise promoter demethylation from precursor to metastasis	Inverse correlation of BORIS with ERα, PR and AR expression; CTCF mutations in ~13% of cases further relieve BORIS silencing
**Prostate**	[40]	Clinical	Prostate cancer patient tissue	BORIS absent in benign prostatic epithelium; cancer-specific expression; positively correlates with Gleason score and T-stage	First demonstration linking BORIS expression to tumour grade and invasiveness in prostate cancer
**Prostate**	[41]	Expression/epigenetic	Prostate cancer cell lines and tissue	Elevated BORIS in individual tumour samples; coincides with promoter hypomethylation	5-aza-2′-deoxycytidine markedly reactivates BORIS, demonstrating that DNA methylation can control BORIS transcription

Abbreviations: EOC, epithelial ovarian cancer; HGSC, high-grade serous carcinoma; FTSECs, fallopian tube secretory epithelial cells; KD, knockdown; KO, knockout; OS, overall survival; PFS, progression-free survival; RFS, recurrence-free survival; CTA, cancer-testis antigen; CSC, cancer stem cell; EMT, epithelial-to-mesenchymal transition; AR, androgen receptor; ERα, oestrogen receptor alpha; PR, progesterone receptor.

## Data Availability

No new data were created or analyzed in this study. Data sharing is not applicable to this article.

## References

[1] Bray F., Laversanne M., Sung H., Ferlay J., Siegel R.L., Soerjomataram I., Jemal A. (2024). Global cancer statistics 2022: GLOBOCAN estimates of incidence and mortality worldwide for 36 cancers in 185 countries. CA Cancer J. Clin..

[2] Boye A., Osei S.A., Brah A.S. (2024). Therapeutic prospects of sex hormone receptor signaling in hormone-responsive cancers. Biomed. Pharmacother..

[3] Ascione L., Castellano G., Curigliano G., Zagami P. (2024). Endocrine therapy for early breast cancer in the era of oral selective estrogen receptor degraders: Challenges and future perspectives. Curr. Opin. Oncol..

[4] Huang L., He W., Guo Y. (2025). From Androgen Deprivation to Precision Therapy: A Bibliometric Review of Global Research Trends (2008–2023). Am. J. Mens Health.

[5] Vellky J.E., Ricke W.A. (2020). Development and prevalence of castration-resistant prostate cancer subtypes. Neoplasia.

[6] Shao B., Zeng Y. (2025). Mechanisms of neuroendocrine differentiation in castration-resistant prostate cancer and advances in the treatment of neuroendocrine prostate cancer. Ann. Med..

[7] Hartkopf A.D., Grischke E.M., Brucker S.Y. (2020). Endocrine-Resistant Breast Cancer: Mechanisms and Treatment. Breast Care.

[8] Hanahan D. (2022). Hallmarks of Cancer: New Dimensions. Cancer Discov..

[9] Suzuki T., Kosaka-Suzuki N., Pack S., Shin D.M., Yoon J., Abdullaev Z., Pugacheva E., Morse H.C., Loukinov D., Lobanenkov V. (2010). Expression of a testis-specific form of Gal3st1 (CST), a gene essential for spermatogenesis, is regulated by the CTCF paralogous gene BORIS. Mol. Cell. Biol..

[10] Pugacheva E.M., Suzuki T., Pack S.D., Kosaka-Suzuki N., Yoon J., Vostrov A.A., Barsov E., Strunnikov A.V., Morse H.C., Loukinov D. (2010). The structural complexity of the human BORIS gene in gametogenesis and cancer. PLoS ONE.

[11] The UniProt Consortium (2025). UniProt: The Universal Protein Knowledgebase in 2025. Nucleic Acids Res..

[12] Renaud S., Pugacheva E.M., Delgado M.D., Braunschweig R., Abdullaev Z., Loukinov D., Benhattar J., Lobanenkov V. (2007). Expression of the CTCF-paralogous cancer-testis gene, brother of the regulator of imprinted sites (BORIS), is regulated by three alternative promoters modulated by CpG methylation and by CTCF and p53 transcription factors. Nucleic Acids Res..

[13] Pugacheva E.M., Bhatt D.N., Rivero-Hinojosa S., Tajmul M., Fedida L., Price E., Ji Y., Loukinov D., Strunnikov A.V., Ren B. (2024). BORIS/CTCFL epigenetically reprograms clustered CTCF binding sites into alternative transcriptional start sites. Genome Biol..

[14] Debruyne D.N., Dries R., Sengupta S., Seruggia D., Gao Y., Sharma B., Huang H., Moreau L., McLane M., Day D.S. (2019). BORIS promotes chromatin regulatory interactions in treatment-resistant cancer cells. Nature.

[15] Hore T.A., Deakin J.E., Marshall Graves J.A. (2008). The evolution of epigenetic regulators CTCF and BORIS/CTCFL in amniotes. PLoS Genet..

[16] Pugacheva E.M., Kubo N., Loukinov D., Tajmul M., Kang S., Kovalchuk A.L., Strunnikov A.V., Zentner G.E., Ren B., Lobanenkov V.V. (2020). CTCF mediates chromatin looping via N-terminal domain-dependent cohesin retention. Proc. Natl. Acad. Sci. USA.

[17] Lutz M., Burke L.J., Barreto G., Goeman F., Greb H., Arnold R., Schultheiss H., Brehm A., Kouzarides T., Lobanenkov V. (2000). Transcriptional repression by the insulator protein CTCF involves histone deacetylases. Nucleic Acids Res..

[18] Kim S., Yu N.K., Kaang B.K. (2015). CTCF as a multifunctional protein in genome regulation and gene expression. Exp. Mol. Med..

[19] Renaud S., Loukinov D., Alberti L., Vostrov A., Kwon Y.W., Bosman F.T., Lobanenkov V., Benhattar J. (2011). BORIS/CTCFL-mediated transcriptional regulation of the hTERT telomerase gene in testicular and ovarian tumor cells. Nucleic Acids Res..

[20] Pugacheva E.M., Teplyakov E., Wu Q., Li J., Chen C., Meng C., Liu J., Robinson S., Loukinov D., Boukaba A. (2016). The cancer-associated CTCFL/BORIS protein targets multiple classes of genomic repeats, with a distinct binding and functional preference for humanoid-specific SVA transposable elements. Epigenet. Chromatin.

[21] Salgado-Albarran M., Gonzalez-Barrios R., Guerra-Calderas L., Alcaraz N., Estefania Sanchez-Correa T., Castro-Hernandez C., Sanchez-Perez Y., Arechaga-Ocampo E., Garcia-Carranca A., Cantu de Leon D. (2019). The epigenetic factor BORIS (CTCFL) controls the androgen receptor regulatory network in ovarian cancer. Oncogenesis.

[22] Loukinov D.I., Pugacheva E., Vatolin S., Pack S.D., Moon H., Chernukhin I., Mannan P., Larsson E., Kanduri C., Vostrov A.A. (2002). BORIS, a novel male germ-line-specific protein associated with epigenetic reprogramming events, shares the same 11-zinc-finger domain with CTCF, the insulator protein involved in reading imprinting marks in the soma. Proc. Natl. Acad. Sci. USA.

[23] Jelinic P., Stehle J.C., Shaw P. (2006). The testis-specific factor CTCFL cooperates with the protein methyltransferase PRMT7 in H19 imprinting control region methylation. PLoS Biol..

[24] Liao J., Song S., Gusscott S., Fu Z., VanderKolk I., Busscher B.M., Lau K.H., Brind’Amour J., Szabo P.E. (2023). Establishment of paternal methylation imprint at the H19/Igf2 imprinting control region. Sci. Adv..

[25] Sleutels F., Soochit W., Bartkuhn M., Heath H., Dienstbach S., Bergmaier P., Franke V., Rosa-Garrido M., van de Nobelen S., Caesar L. (2012). The male germ cell gene regulator CTCFL is functionally different from CTCF and binds CTCF-like consensus sites in a nucleosome composition-dependent manner. Epigenet. Chromatin.

[26] Li X.F., Ren P., Shen W.Z., Jin X., Zhang J. (2020). The expression, modulation and use of cancer-testis antigens as potential biomarkers for cancer immunotherapy. Am. J. Transl. Res..

[27] Gjerstorff M.F., Andersen M.H., Ditzel H.J. (2015). Oncogenic cancer/testis antigens: Prime candidates for immunotherapy. Oncotarget.

[28] Marshall A.D., Bailey C.G., Champ K., Vellozzi M., O’Young P., Metierre C., Feng Y., Thoeng A., Richards A.M., Schmitz U. (2017). CTCF genetic alterations in endometrial carcinoma are pro-tumorigenic. Oncogene.

[29] Woloszynska-Read A., Zhang W., Yu J., Link P.A., Mhawech-Fauceglia P., Collamat G., Akers S.N., Ostler K.R., Godley L.A., Odunsi K. (2011). Coordinated cancer germline antigen promoter and global DNA hypomethylation in ovarian cancer: Association with the BORIS/CTCF expression ratio and advanced stage. Clin. Cancer Res..

[30] Hillman J.C., Pugacheva E.M., Barger C.J., Sribenja S., Rosario S., Albahrani M., Truskinovsky A.M., Stablewski A., Liu S., Loukinov D.I. (2019). BORIS Expression in Ovarian Cancer Precursor Cells Alters the CTCF Cistrome and Enhances Invasiveness Through GALNT14. Mol. Cancer Res..

[31] Salgado-Albarran M., Spath J., Gonzalez-Barrios R., Baumbach J., Soto-Reyes E. (2022). CTCFL regulates the PI3K-Akt pathway and it is a target for personalized ovarian cancer therapy. npj Syst. Biol. Appl..

[32] Alberti L., Renaud S., Losi L., Leyvraz S., Benhattar J. (2014). High expression of hTERT and stemness genes in BORIS/CTCFL positive cells isolated from embryonic cancer cells. PLoS ONE.

[33] D’Arcy V., Abdullaev Z.K., Pore N., Docquier F., Torrano V., Chernukhin I., Smart M., Farrar D., Metodiev M., Fernandez N. (2006). The potential of BORIS detected in the leukocytes of breast cancer patients as an early marker of tumorigenesis. Clin. Cancer Res..

[34] D’Arcy V., Pore N., Docquier F., Abdullaev Z.K., Chernukhin I., Kita G.X., Rai S., Smart M., Farrar D., Pack S. (2008). BORIS, a paralogue of the transcription factor, CTCF, is aberrantly expressed in breast tumours. Br. J. Cancer.

[35] Dougherty C.J., Ichim T.E., Liu L., Reznik G., Min W.P., Ghochikyan A., Agadjanyan M.G., Reznik B.N. (2008). Selective apoptosis of breast cancer cells by siRNA targeting of BORIS. Biochem. Biophys. Res. Commun..

[36] Alberti L., Losi L., Leyvraz S., Benhattar J. (2015). Different Effects of BORIS/CTCFL on Stemness Gene Expression, Sphere Formation and Cell Survival in Epithelial Cancer Stem Cells. PLoS ONE.

[37] Loukinov D., Anderson A.L., Mkrtichyan M., Ghochikyan A., Rivero-Hinojosa S., Tucker J., Lobanenkov V., Agadjanyan M.G., Nelson E.L. (2023). A Therapeutic Vaccine Targeting Rat BORIS (CTCFL) for the Treatment of Rat Breast Cancer Tumors. Int. J. Mol. Sci..

[38] Risinger J.I., Chandramouli G.V., Maxwell G.L., Custer M., Pack S., Loukinov D., Aprelikova O., Litzi T., Schrump D.S., Murphy S.K. (2007). Global expression analysis of cancer/testis genes in uterine cancers reveals a high incidence of BORIS expression. Clin. Cancer Res..

[39] Hoivik E.A., Kusonmano K., Halle M.K., Berg A., Wik E., Werner H.M., Petersen K., Oyan A.M., Kalland K.H., Krakstad C. (2014). Hypomethylation of the CTCFL/BORIS promoter and aberrant expression during endometrial cancer progression suggests a role as an Epi-driver gene. Oncotarget.

[40] Cheema Z., Hari-Gupta Y., Kita G.X., Farrar D., Seddon I., Corr J., Klenova E. (2014). Expression of the cancer-testis antigen BORIS correlates with prostate cancer. Prostate.

[41] Hoffmann M.J., Muller M., Engers R., Schulz W.A. (2006). Epigenetic control of CTCFL/BORIS and OCT4 expression in urogenital malignancies. Biochem. Pharmacol..

[42] Pugacheva E.M., Rivero-Hinojosa S., Espinoza C.A., Mendez-Catala C.F., Kang S., Suzuki T., Kosaka-Suzuki N., Robinson S., Nagarajan V., Ye Z. (2015). Comparative analyses of CTCF and BORIS occupancies uncover two distinct classes of CTCF binding genomic regions. Genome Biol..

[43] Hnisz D., Weintraub A.S., Day D.S., Valton A.L., Bak R.O., Li C.H., Goldmann J., Lajoie B.R., Fan Z.P., Sigova A.A. (2016). Activation of proto-oncogenes by disruption of chromosome neighborhoods. Science.

[44] Zhou Y., Gerrard D.L., Wang J., Li T., Yang Y., Fritz A.J., Rajendran M., Fu X., Stein G., Schiff R. (2019). Temporal dynamic reorganization of 3D chromatin architecture in hormone-induced breast cancer and endocrine resistance. Nat. Commun..

[45] Achinger-Kawecka J., Valdes-Mora F., Luu P.L., Giles K.A., Caldon C.E., Qu W., Nair S., Soto S., Locke W.J., Yeo-Teh N.S. (2020). Epigenetic reprogramming at estrogen-receptor binding sites alters 3D chromatin landscape in endocrine-resistant breast cancer. Nat. Commun..

[46] Lawrenson K., Fonseca M.A.S., Liu A.Y., Segato Dezem F., Lee J.M., Lin X., Corona R.I., Abbasi F., Vavra K.C., Dinh H.Q. (2019). A Study of High-Grade Serous Ovarian Cancer Origins Implicates the SOX18 Transcription Factor in Tumor Development. Cell Rep..

[47] Tuna M., Ju Z., Yoshihara K., Amos C.I., Tanyi J.L., Mills G.B. (2020). Clinical relevance of TP53 hotspot mutations in high-grade serous ovarian cancers. Br. J. Cancer.

[48] Reyes H.D., Devor E.J., Warrier A., Newtson A.M., Mattson J., Wagner V., Duncan G.N., Leslie K.K., Gonzalez-Bosquet J. (2019). Differential DNA methylation in high-grade serous ovarian cancer (HGSOC) is associated with tumor behavior. Sci. Rep..

[49] Link P.A., Zhang W., Odunsi K., Karpf A.R. (2013). BORIS/CTCFL mRNA isoform expression and epigenetic regulation in epithelial ovarian cancer. Cancer Immun..

[50] Al-Shami K., Awadi S., Khamees A., Alsheikh A.M., Al-Sharif S., Ala’ Bereshy R., Al-Eitan S.F., Banikhaled S.H., Al-Qudimat A.R., Al-Zoubi R.M. (2023). Estrogens and the risk of breast cancer: A narrative review of literature. Heliyon.

[51] Bokhman J.V. (1983). Two pathogenetic types of endometrial carcinoma. Gynecol. Oncol..

[52] Yamanaka T., Yoshida H., Shimoi T., Sudo K., Yonemori K. (2026). Endocrine Therapy for Endometrial Carcinoma: Current Evidence, Resistance Mechanisms, and Biomarker-Driven Patient Selection. Curr. Oncol..

[53] Quistini A., Chierigo F., Fallara G., Depalma M., Tozzi M., Maggi M., Jannello L.M.I., Pellegrino F., Mantica G., Terracciano D. (2025). Androgen Receptor Signalling in Prostate Cancer: Mechanisms of Resistance to Endocrine Therapies. Res. Rep. Urol..

[54] Li L., Sun Y. (2024). Circulating tumor DNA methylation detection as biomarker and its application in tumor liquid biopsy: Advances and challenges. MedComm.

[55] Cheever M.A., Allison J.P., Ferris A.S., Finn O.J., Hastings B.M., Hecht T.T., Mellman I., Prindiville S.A., Viner J.L., Weiner L.M. (2009). The prioritization of cancer antigens: A national cancer institute pilot project for the acceleration of translational research. Clin. Cancer Res..

[56] Loukinov D. (2018). Targeting CTCFL/BORIS for the immunotherapy of cancer. Cancer Immunol. Immunother..

[57] Jiang J., Li L., Yu Z., Wang K., Ma J., Li D., Guo Z., Wang L., Zhang H., Xu L. (2025). Clinical efficacy and challenges of cancer-testis antigen vaccines in advanced solid tumors: A systematic review and meta-analysis. Crit. Rev. Oncol. Hematol..

[58] Negri A., Marozzi M., Trisciuoglio D., Rotili D., Mai A., Rizzi F. (2023). Simultaneous administration of EZH2 and BET inhibitors inhibits proliferation and clonogenic ability of metastatic prostate cancer cells. J. Enzyme Inhib. Med. Chem..

